# Convection-Enhanced Delivery *In Silico* Study for Brain Cancer Treatment

**DOI:** 10.3389/fbioe.2022.867552

**Published:** 2022-05-25

**Authors:** Chryso Lambride, Vasileios Vavourakis, Triantafyllos Stylianopoulos

**Affiliations:** ^1^ Department of Mechanical and Manufacturing Engineering, University of Cyprus, Nicosia, Cyprus; ^2^ Department of Medical Physics and Biomedical Engineering, University College London, London, United Kingdom

**Keywords:** targeted therapy, drug transport, simulation, mathematical model, pharmacodynamics, drug distribution prediction, finite element method

## Abstract

Brain cancer therapy remains a formidable challenge in oncology. Convection-enhanced delivery (CED) is an innovative and promising local drug delivery method for the treatment of brain cancer, overcoming the challenges of the systemic delivery of drugs to the brain. To improve our understanding about CED efficacy and drug transport, we present an *in silico* methodology for brain cancer CED treatment simulation. To achieve this, a three-dimensional finite element formulation is utilized which employs a brain model representation from clinical imaging data and is used to predict the drug deposition in CED regimes. The model encompasses biofluid dynamics and the transport of drugs in the brain parenchyma. Drug distribution is studied under various patho-physiological conditions of the tumor, in terms of tumor vessel wall pore size and tumor tissue hydraulic conductivity as well as for drugs of various sizes, spanning from small molecules to nanoparticles. Through a parametric study, our contribution reports the impact of the size of the vascular wall pores and that of the therapeutic agent on drug distribution during and after CED. The *in silico* findings provide useful insights of the spatio-temporal distribution and average drug concentration in the tumor towards an effective treatment of brain cancer.

## Introduction

Treatment of brain cancer remains a challenge despite the recent significant improvements of several modalities, such as chemotherapy, immunotherapy, and targeted therapy with nanomedicines (Shi and Sanche, 2019). Numerous obstacles are associated with the treatment of neurological diseases; one of the most significant being the blood-brain barrier (BBB), which is in large part responsible for the gap between scientific progress and improved treatment outcomes (Mehta et al., 2017). BBB exists along the cerebral capillaries and isolates the systemic circulation from the cerebral parenchyma, providing protection to brain cells. Apart from its protective role, BBB hinders the systemic delivery of therapeutics into brain tumors (Koo et al., 2006; Li et al., 2010; Mehta et al., 2017; Arvanitis et al., 2020). However, during tumor progression, the tumor vasculature becomes increasingly heterogeneous and abnormal, BBB is disrupted, and tumor vessels are generally considered much leakier than the healthy ones. Therefore, in the case where a drug is administered intravenously, a higher drug concentration is accumulated within brain tumors as compared with the unaffected brain (Li et al., 2010; Arvanitis et al., 2020). Similar to other tumor types, tumor vessel hyper-permeability results in heterogeneous transvascular transport of small and large molecules as well as heterogeneous perfusion, contributing to suboptimal drug accumulation in brain tumor, and making systemic delivery methods highly ineffective (Lueshen et al., 2017). Innovative therapies have been recently developed, overcoming the challenges posed by the BBB with limited systemic toxicity and achieving promising results in the effective treatment of brain cancer. The basic principle of these strategies is to deliver and achieve high concentration of the drugs in the desired areas of the brain by bypassing the BBB in various ways. Generally, the advanced therapeutic approaches are classified into invasive and non-invasive, and present selective advantages and limitations (Koo et al., 2006; Ferguson et al., 2007; Juratli et al., 2013; Pandit et al., 2020).

Convection-enhanced delivery (CED) is such a promising method for localized delivery of drugs to brain tumors. The technique was suggested as a method to transfer drugs, which are either limited by the BBB or are too large to diffuse effectively (Bobo et al., 1994; Raghavan et al., 2006; Mehta et al., 2017). CED involves a minimal invasive surgical exposure of the brain (Pandit et al., 2020) and can bypass the BBB by direct drug infusion into the interstitial space of the brain tumor *via* surgically placed catheters that reach the peritumoral region and enter the tumor (Shi and Sanche, 2019; Pandit et al., 2020). Therefore, CED offers important advantages over systemic chemotherapy (Koo et al., 2006; Tykocki and Miekisiak, 2016). CED utilizes a positive pressure gradient created by an infusion pump to inject drugs through the catheter into the interstitial space of the solid tumor tissue. Thus, a diverse type of therapeutic agents can be administered directly to a specific targeted area, enabling the distribution of large volumes of high drug concentrations with minimum systemic toxicity. Additionally, application of this drug delivery method can lead to a fast coverage of large tumor volumes and to the reduction of possible side effects (Ferguson et al., 2007; Allard et al., 2009; Juratli et al., 2013; Tykocki and Miekisiak, 2016; Lueshen et al., 2017; Pandit et al., 2020). To extensively investigate drug delivery techniques, mathematical and computational models have been developed allowing for well-controlled studies which would not be possible or economically viable through experiments (Zhan et al., 2018). Consequently, *in silico* cancer modeling has demonstrated great potential as a tool to simulate the drug transport and delivery in solid tumors and optimize the delivery conditions to desired sites and thus, improve therapeutic efficacy and treatment outcome (Hadjicharalambous et al., 2021).

Several *in silico* studies have been developed to understand better the mechanism and limitations of CED method. Støverud et al. (2012) created a numerical model which includes both drug transport and tissue expansion using diffusion tensor imaging (DTI) data. Due to tissue swelling during CED infusion, a poroelasticity theory has been incorporated into several mathematical models to describe changes in the hydraulic tissue environment, in terms of porosity and permeability (Raghavan et al., 2006; Raghavan and Brady, 2011; Støverud et al., 2012). A more recent similar approach carried out both *in silico* and *in vivo* investigations for two human clinical trials of immunotoxins, using different tracer molecules (Brady et al., 2020). This study concluded that crucial parameters of flow include infusion-induced tissue expansion and loss through vessel walls. Other computational studies have been employed to investigate a catheter design and placement, infusion flow rate and how drug distribution, backflow and reflux are affected (Lueshen et al., 2017; Antoine et al., 2020; Orozco et al., 2020), providing guidelines for effective CED (Antoine et al., 2020). Studies also focused on the engineering of a novel backflow-free catheter, allowing the therapeutics to reach an increased concentration to the site of delivery and a more predictable distribution that is critical for patient care (Lueshen et al., 2017). In addition, Linninger et al. (2008) suggested a concise tool for selecting suitable infusion and catheter design parameters systematically based on advanced imaging techniques and experimental data, maximizing penetration depth and volumes of distribution in the desired region. They found that regional and structural heterogeneity of the brain tissue influence drug distribution. The predictions were confirmed with experimental trials, indicating that for a given flow rate, thinner catheters lead to larger distribution volumes (Linninger et al., 2008). Zhan and co-workers carried out a series of computational and mathematical studies. They examined drug transport under different CED operating conditions, i.e., infusion rate, solution concentration and infusion site location. Their modeling predictions suggested that drug penetration can be improved by raising the infusion rate and the infusion solution concentration, and high drug concentrations can be achieved mainly around the infusion site (Zhan et al., 2017). A year later, Zhan et al. (2017) studied CED of six chemotherapeutic drugs based on a multi-physical model. They concluded that the drug non-uniform penetration and accumulation in the brain tumor are strongly dependent on its physicochemical properties (Zhan and Wang, 2018a). In the same year, Zhan and co-workers investigated the CED of liposome encapsulated doxorubicin under various delivery conditions (Zhan and Wang, 2018b). They found that compared to the direct infusion of doxorubicin, the drug accumulation and penetration can be enhanced by using liposome-CED method. This treatment can be improved by either increasing the liposome solution concentration and infusion rate, decreasing the liposome vascular permeability, or placing the infusion site in tumor with sparse microvasculature (Zhan and Wang, 2018b). Zhan et al. (2019) conducted additional studies for the effects of tissue permeability and drug diffusion anisotropy on the CED of different drugs. They proposed that the anisotropy tissue permeability affects insignificantly the effective delivery volume, however it can alter the drug spatial distribution (Zhan et al., 2019). Furthermore, Zhan et al. (2019) investigated the effectiveness of various cytotoxic drugs in the combination with anti-angiogenic treatment (Zhan, 2020). Predictions showed that combination of chemotherapy with anti-angiogenesis could enhance delivery of all drugs examined using CED administration (Zhan, 2020).

Motivated by the previous studies, we propose here three-dimensional (3D) finite element (FE) model of CED for the treatment of brain cancer. The 3D FE model incorporates: i) biofluid mechanics for the fluid pressure and velocity distributions in the tumor, ii) diluted species transport equations for the description of the distribution of the drug in the tumor and peritumoral area, and iii) the theory for hindered transport of rigid solutes through liquid filled pores to describe the transvascular transport of drugs across the tumor vessel walls, taking explicitly into account the drug size and the pore size of the tumor vascular walls. However, a succinct comparison of the modelling features of this manuscript against recent previous works is provided in Supplementary Table S1. Here, we aim to study the drug concentration during and after CED administration of different drug sizes by changing the patho-physiological conditions of the tumor tissue. To achieve this, therapeutic agents of 1, 20, and 60 nm in diameter, and vessel wall pores of tumor tissue with diameters 50, 100, and 150 nm have been considered. Subsequently, changes in these sizes affect both the vascular characteristics (e.g., vascular hydraulic conductivity) and the drug properties (e.g., drug transvascular permeability and diffusion coefficient). More specifically, the vessel wall pore size defines the vascular hydraulic conductivity, whereas the relative size of the drug to the vessel wall pore size determines the permeability of the drug across the vessels. This is important because structural abnormalities in the tumor vasculature (that cause vessel hyper-permeability) is a hallmark of tumor patho-physiology and brain tumors is not an exception. So, despite the BBB effect, abnormal brain tumor vessels can have large openings/pores. Furthermore, the impact of the hydraulic conductivity of the tumor interstitial space on the distribution of the drug is extensively investigated.

## Materials and Methods

### Three-Dimensional Reconstruction and Model Generation From Clinical Image Data

**FIGURE 1:**
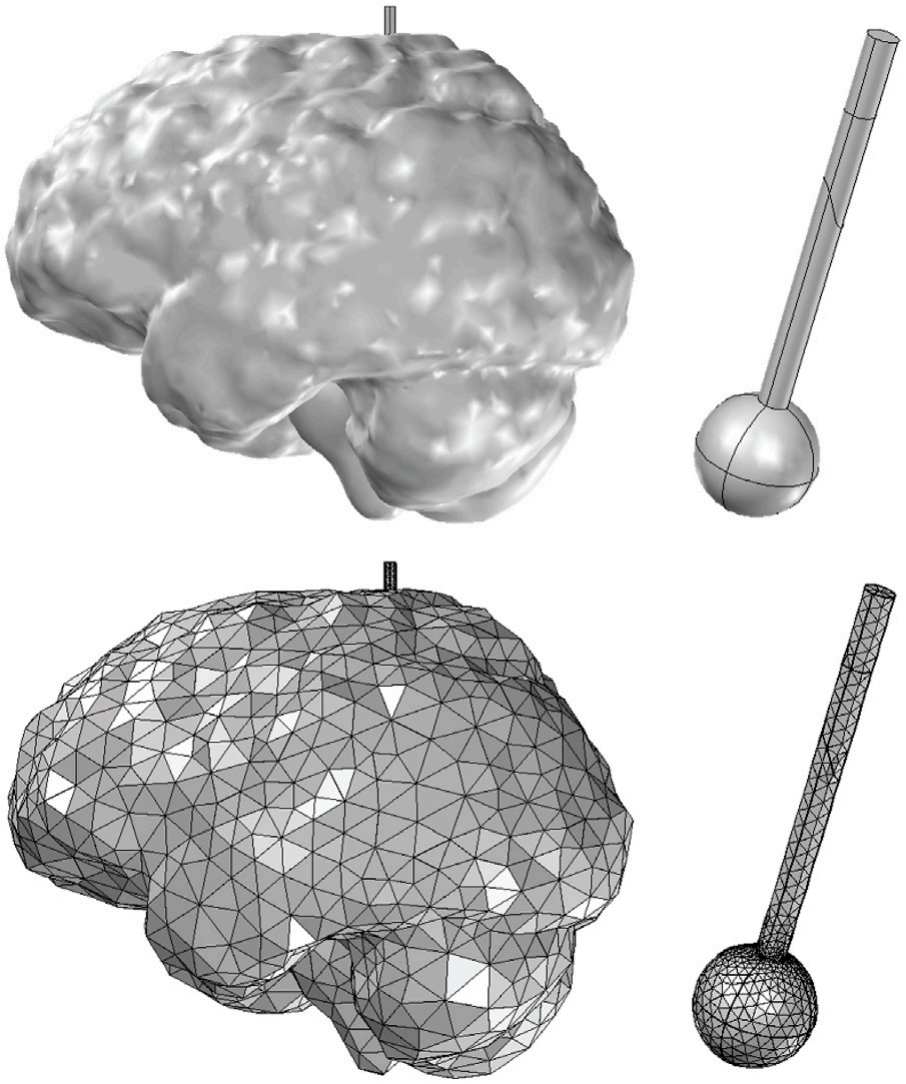
Three-dimensional domain and FE model employed for simulating convection-enhanced delivery of drugs to brain tumors. The models consist of the white and gray matter of the brain reconstructed from MR images of a healthy adult, a tumor of spherical shape (6 mm radius) and the catheter (1.5 mm radius). The entire model consists of 88,530 FEs.

Magnetic resonance (MR) images of a healthy adult subject were used to create realistic FE model of the brain in 3D. The MR images were acquired from our previous study (Angeli and Stylianopoulos, 2016) and were reused in this work. Specifically, for the morphological imaging of the brain a T1-weighted, three-dimensional, fast field echo pulse sequence was acquired with an echo and repetition time of 3.2 and 7.1 ms respectively, while an isotropic voxel size of 1 mm was used to cover the entire brain. The commercial software ScanIP from Simpleware (version 6.0; Synopsys, Mountain View, United States) was employed for the three-dimensional reconstruction of the brain geometry. Specifically, two masks were first generated from the MR images using Simpleware’s “threshold” operation, which selects each pixel according to its brightness. The darkest areas comprise the mask of the gray matter, whereas the brightest regions comprise the mask of the white matter in the brain. These masks were the two different domains of the resulting 3D brain geometry. Then, the “island removal” and “cavity fill” operations were used to eliminate small unconnected parts of the masks and fill any gaps of the model, respectively. Additionally, smoothing was performed using Simpleware’s “Gaussian smoothing” operation. The 3D brain geometry was eventually created and exported in a COMSOL-compatible geometry file. The 3D brain geometry was imported to the commercial FE software COMSOL Multiphysics (version 5.5; COMSOL Inc., Burlington, MA, United States). Subsequently, a sphere with radius 6 mm and a cylinder with radius 1.5 mm were formed inside the brain geometry to represent the tumor and catheter domains, respectively. The sphere (brain tumor) was located in the white matter inside the parietal lobe of the left cerebral hemisphere. Hence, the 3D brain geometry consisted of four domains, i.e., gray matter, white matter, tumor, and catheter. Next, using COMSOL Multiphysics software, the FE mesh was created though the “free tetrahedral” and “boundary layer” operations to form an optimal mesh with boundary layers at the interfaces between the geometry domains. Figure 1 illustrates the individualized 3D FE brain model used for the CED simulations.

### Mathematical Model

The brain tissue was modeled as a porous medium (Angeli and Stylianopoulos, 2016) and the drug distribution and pharmacodynamics model was based on the conservation equations of mass transport and momentum balance. The drugs were released directly into the tumor interstitial space through the catheter and then they could travel inside the tumor by convection and diffusion, exit the tumor region towards the surrounding healthy tissue and be cleared by tumor and healthy vessels by convective and diffusive mechanisms.

Interstitial flow within the porous brain tissue obeys to the continuity equation and the extended Darcy’s law (Nield and Bejan, 1999). The continuity equation of tumor fluid phase is given by:

$$\nabla \cdot u=Q_{m},$$ (1)

where ***u*** is interstitial fluid velocity (IFV) and *Q*_*m*_ is the fluid flux that is exchanged between the tissue and the vascular system, and it is mathematically modelled using Starling’s approximation (Vavourakis et al., 2017; Lambride et al., 2020):

$$Q_{m}=L_{p}S_{v}\left(p_{v}-p_{i}\right)-L_{pl}S_{vl}\left(p_{i}-p_{l}\right),$$ (2)

where *L*_*p*_, *S*_*v,*_ and *p*_*v*_ are the hydraulic conductivity of the vessel wall, the vascular density, and the vascular pressure of the blood vessels respectively, whereas *L*_*pl*_, *S*_*vl*_, and *p*_*l*_ are the corresponding quantities for the lymphatic vessels, and *p*_*i*_ is the interstitial fluid pressure (IFP). The first term in Eq. 2 refers to the fluid flux entering the tumor or the surrounding normal tissue from the blood vessels, while the second term refers to the fluid flux exiting through the lymphatic system. It is important to note that the second term was set to zero in the tumor tissue due to the dysfunctional lymphatic system (Stylianopoulos et al., 2018).

We extended Darcy’s law due to the presence of infusate flow from the CED catheter, to describe the fluid motion in porous tissues of the brain (Nield and Bejan, 1999; Linninger et al., 2008). The Brinkman equation extends Darcy’s law to describe the dissipation of the kinetic energy by viscous shear, similar to the Navier-Stokes equation. Thus, the momentum balance is given by:

$$\rho \frac{\partial u}{\partial t}=\nabla \cdot \left[\frac{\mu_{i}}{\varepsilon_{p}}\left(\nabla \mathrm{u+u}\nabla \right)-\left(\frac{2}{3}\frac{\mu }{\varepsilon_{p}}\nabla \cdot u+P_{i}\right)I\right]-\left(\frac{1}{k}+\frac{Q_{m}}{\varepsilon_{p^{2}}}\right)u,$$ (3)

where *ε*_*p*_ is the tissue porosity, *k* is the hydraulic conductivity of the interstitial space, *μ*_*i*_ and *ρ* refer to the viscosity and the density of interstitial fluid, respectively.

Regarding the drug concentration in the porous brain tissue, the convection-diffusion mass transport equation was applied, incorporating the exchange of drug between tissue and the blood vessels with the term *Q* (Mpekris et al., 2017):

$$\frac{\partial C_{i}}{\partial t}+\nabla \cdot \left(C_{i}u-D_{i}\nabla C_{i}\right)=Q-k_{d}C_{i}-k_{l}C_{i},$$ (4)

where *C*_*i*_ refers to the relative concentration of drug in the interstitial space, which is a dimensionless quantity, i.e., the ratio of the local drug concentration to the value of the concentration entering the catheter, and *D*_*i*_ is the diffusion coefficient of the drug in the interstitial space. Additionally, *k*_*d*_ is the drug degradation rate constant, while $K_{l}$ is the lymphatic drainage constant, applicable only in the healthy tissue region (Linninger et al., 2008; Stylianopoulos et al., 2018).

The total net transvascular drug flux between the brain tissue and the vessels can be written neglecting the oncotic pressure difference across the wall (Mpekris et al., 2017; Stylianopoulos et al., 2018):

$$Q=\left(C_{v}-C_{i}\right)PS_{v}+\left(1-\sigma_{f}\right)\left(p_{v}-p_{i}\right)L_{p}S_{v}C_{v},$$ (5)

where *p* is the vascular permeability of the drug through the pores of the vessel wall, *C*_*v*_ is the vascular concentration of the drug, and *σ*_*f*_ is the reflection coefficient. In CED, the therapeutic agents are injected directly inside the brain tumor tissue through a catheter; thereby in the tumor domain the vascular drug concentration (*C*_*v*_) is considered negligible compared to the interstitial space drug concentration (*C*_*i*_).

The therapeutic agent was considered to be of spherical shape and without any surface charge and the vessel wall openings were modeled as cylindrical pores (Deen, 1987). To investigate the direct effect of the vascular wall pore size and drug size on the drug concentration distribution, the theory for hindered transport of rigid solutes through liquid filled porous was introduced. Using this theory, the hydraulic conductivity of vascular walls, *L*_*p*_, the drug vascular permeability, *p*, and the reflection coefficient, *σ*_*f*_, were explicitly estimated based on the ratio of particle size to the vessel wall pore size. The hydraulic conductivity of vessel walls is calculated *via* Eq. 6 and the vascular permeability of the drug is defined by Eq. 7:

$$L_{p}=\frac{\gamma r_{0}^{2}}{8\mu L_{vw}},$$ (6)

$$P=\frac{\gamma HD}{L_{vw}},$$ (7)

where *γ* is the fraction of vessel all surface area occupied by pores, *r*_*o*_ is the pore radius, *μ* is the viscosity of plasma at 310 K, *L*_*vw*_ is the thickness of the vessel wall, and *H* corresponds to the hydrodynamic coefficient for neutral spheres in cylindrical pores. *D* is the diffusion coefficient of a particle with radius *r*_*s*_, given by the Stokes-Einstein relationship, $D=\frac{K_{b}T}{6\pi \mu r_{s}}$ , where *K*_*b*_ is the Boltzmann constant, and *T* is the temperature of the solution (Deen, 1987). Definition of the reflection coefficient, *σ*_*f*_, and the hydrodynamic coefficient, *H*, is provided in Supplementary Table S2.

### Model Parameters Values and Boundary Conditions

For a realistic 3D finite element (FE) brain model, data from experimental studies were used to determine the physiological and biomechanical properties. As in pertinent studies, the diameters of the vessel wall pores were set to 50, 100, and 150 nm (Hobbs et al., 1998; Sarin et al., 2009; Chauhan et al., 2012), whereas the hydraulic conductivity of the brain tumor interstitial space, *k*, has been reported to vary significantly, as a result it was taken equal to 2 × 10^−14^, 2 × 10^−13^, and 2 × 10^−12^ m^2^/(Pa s) (Netti et al., 2000; Smith and Humphrey, 2007). Optimal size of nanoparticles for cancer treatment ranges from 20 to 70 nm in diameter to easily diffuse into tumor ECM (Koo et al., 2006; Vavourakis et al., 2018; Wijeratne and Vavourakis, 2019). Recent studies have showed that particles of sizes 40–50 nm are able to effectively bind and induce receptor-mediated endocytic processes (Jain and Stylianopoulos, 2010). Additionally, it has been shown that particles larger than 60 nm in diameter are less effective diffusing through the extracellular matrix space (Jain and Stylianopoulos, 2010). With this in mind, we decided to focus on the drug-size range of 1–60 nm in diameter referring to conventional chemotherapeutics (<2 nm), antibodies and liposomes. For a relevant drug-size comparison summary for brain tumors, the reader should refer to Table 1 from (Koo et al., 2006). Thus, the effective diameter of the therapeutic agents varied from 1 nm for small-size molecules, to 20 and 60 nm for liposomes to investigate the delivery of a wide range of drug sizes. All values of the model parameters are summarized in Supplementary Table S3. It is noteworthy that for patient-specific studies, the parameters in Eq. 5 can be estimated for a small drug by direct measurement in a DCE imaging protocol to accurately define the drug capillary loss rate (Brady et al., 2020).

For each geometry domain, the conservation equations of mass and momentum were discretized and solved numerically coupled using the commercial FE software COMSOL Multiphysics 5.5 (COMSOL Inc., Burlington, MA, United States). The Brinkman equation module of the FE software was used, providing the conservation of mass and momentum that can fully describe the fluid dynamics within each geometry domains. The Brinkman equation module computes both the velocity filed and pressure, which are the dependent variables. Additionally, both the velocity (vector) field and pressure (scalar) were discretized using linear Lagrange basis functions. Likewise, the drug concentration (scalar) was discretized using linear Lagrange basis functions. COMSOL project files for two representative simulation cases of CED can be freely accessed on Figshare: https://figshare.com/projects/Convection-Enhanced_Delivery_in_silico_study_for_personalized_brain_cancer_treatment/135923.

Following data from relevant clinical CED studies (Barua et al., 2013; Shi and Sanche, 2019; Stine and Munson, 2019; Tosi and Souweidane, 2020), the infusion flow rate, *Q*_*f*_, ranged from 0.025 to 0.75 ml/h and the infusion volume varied between 0.25 and 185 ml. A flow rate of 0.5 ml/h and an infusion volume of 3 ml were selected for all simulations. Therefore, at the interface between the catheter and tumor the normal inlet velocity (i.e., *U*_*o*_ *=Q*_*f*_ */A*, where *A* the cross section of the catheter) was taken equal to 1.99 × 10^−5^ m/s and infusion lasted for 6 h. Also, at the interface of the catheter and tumor tissue, the relative drug concertation was set to unity for the period of the infusion and after completion of infusion, a zero-flux boundary condition was applied (i.e., $n\cdot \left(D\nabla C_{i}-uC_{i}\right)=0$ *,* where ***n*** corresponds to the outward unit normal vector).The normal stresses on the outer brain surfaces were equal to zero (i.e., *n σ = 0*). At the catheter surfaces, a no-slip boundary condition was applied for fluid velocity (i.e., *u = 0*) and additionally, a zero-flux boundary condition was set for the transport of the drugs. The latter boundary condition was also set at the outer brain surfaces (Supplementary Figure S1). Regarding initial values, both the fluid velocity and pressure variables were set to zero for all geometry domains. Likewise, the initial value of the drug concentration was zero everywhere in the brain model.

## Results

Numerical investigation of convection-enhanced drug delivery and distribution in the human brain was considered by varying the drug size and the physiological properties of the tumor microenvironment. It is well established that therapeutic agents’ transport can be described through diffusion or/and convection (Stylianopoulos et al., 2018). The latter transport mechanism is owing to pressure gradients, thus, the investigation of IFP and IFV profiles are crucial for understanding the drug delivery mechanism. The drugs were released directly into the tumor interstitial space and then they were allowed to travel inside the tumor, wash out of the tumor to the surrounding healthy tissue and be cleared by the vessels. Also, to accomplish the objectives of this *in silico* study, therapeutic agents of three distinct sizes were selected with hydrodynamic diameter: 1, 20, and 60 nm, spanning the range from small molecules to nanomedicines. The simulations were performed by changing the hydraulic conductivity of the tumor interstitial space (from 2 × 10^−12^ to 2 × 10^−14^ m^2^/(Pa s)) and the vessel wall pore diameter (from 50 to 150 nm). It is important to note that the relative size of the drug to the vessel wall pore size determines the drug permeation through the vessels’ endothelial wall, Eq. 7. Additionally, by increasing the drug size, the diffusion coefficient of the drug through the tumor interstitial space decreases, which has been measured experimentally for macromolecules and particles of various diameters (Pluen et al., 2001; Wijeratne and Vavourakis, 2019). It should be noted that 1) the relative drug concentration (dimensionless quantity) is normalized with respect to the reference value of the drug amount infused through the catheter and 2) average relative concentration (dimensionless quantity) was calculated from the volume integral of the relative drug concentration in the tumor region divided by the tumor volume.

### Interstitial Space Drug Concentration for Baseline Tumor Microenvironment Properties

**FIGURE 2:**
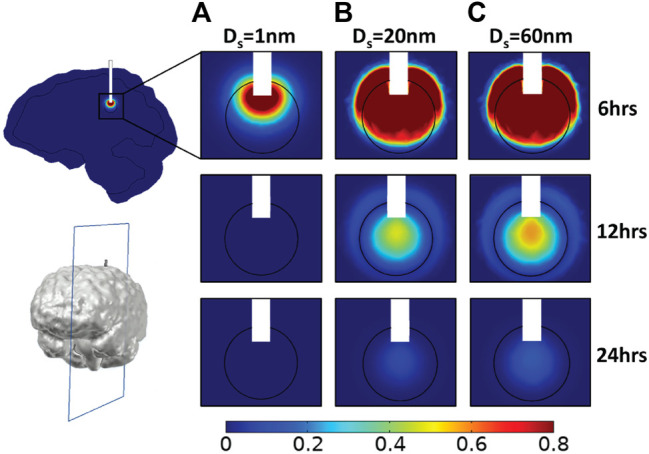
Simulated drug concentration using baseline tumor microenvironment conditions. A sagittal view in the center of tumor tissue showing the spatial distribution of drug concentration and for different diameters of the therapeutic agent, D_s_: **(A)** 1 nm, **(B)** 20 nm, and **(C)** 60 nm at three time points: 6, 12, and 24 h. Drug concentration is normalized by division with the reference value entering the catheter.

**FIGURE 3:**
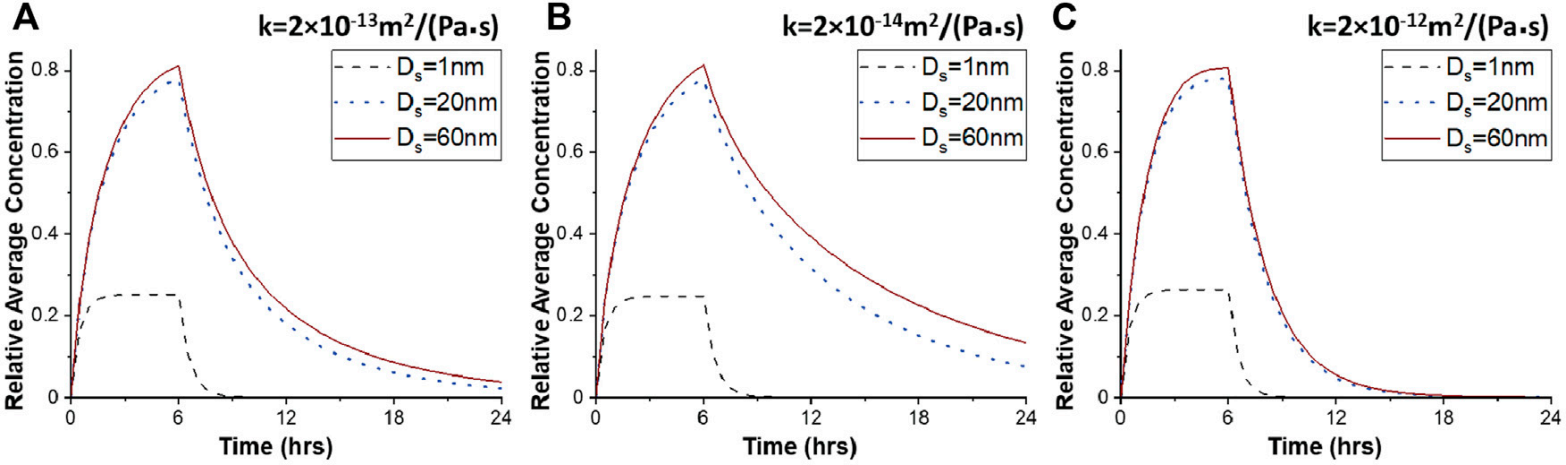
Average drug concentration as a function of the time for different hydraulic conductivities of the tumor interstitial space. Average drug concentration in tumor tissue as a function of time for different diameters of therapeutic agents, D_s_, for 100 nm pore diameter of vascular walls, and for different values of the hydraulic conductivity of the tumor interstitial space, *k*: **(A)** 2 × 10^−13^ m^2^/(Pa s), **(B)** 2 × 10^−14^ m^2^/(Pa s), and **(C)** 2 × 10^−12^ m^2^/(Pa s). Drug concentration is normalized by division with the reference value entering the catheter.

We first set the hydraulic conductivity of the tumor interstitial space equal to 2 × 10^−13^ m^2^/(Pa s) and the diameter of vessel wall pores to 100 nm (baseline tumor microenvironment conditions). Figure 2 illustrates a sagittal view of the center of the tumor tissue, presenting the spatial distribution of different drug sizes in three snapshots, i.e., 6, 12, and 24 h, respectively after CED injection commenced. All spatial drug distributions are symmetric in the vertical axis. In case of the 1 nm drug size diameter, the highest drug concentration during infusion is located near the infusion site. Small molecule therapeutics owing to their small size have a high diffusion coefficient and thus, they travel fast away from the infusion site and can be cleared easily by the pores of the blood vessels. Hence, a significant drug amount is lost because the drug can easily pass into the blood vessels due to concentration gradients. These observations for small drug sizes were also reported by (Brady et al., 2020). Brady et al. (2020) mentioned that the capillary loss rate plays a key role in the drug transport within the tumor tissue, especially for small molecules in which the loss rate is significantly high. As a result, the concentration of the drug in the tumor diminishes soon after the infusion stops (Figure 2A). When the infusion stops, the average relative concentration of the therapeutic agent reaches 0.25 in the tumor tissue, and then decreases sharply to zero (Figure 3A).

For the 20 nm therapeutic agent, vascular permeability becomes 98% lower compared to that of the 1 nm drug size, and thereby, the intravasation of the drug is hindered significantly. This type of nanoparticle is still able to diffuse relatively fast and penetrate the tumor tissue, covering almost the entire tumor region during CED administration (Figure 2B). The maximum average relative concentration of drug in the tumor tissue is 0.78 at the end of the infusion period at 6 h but drug remains in the tumor region for over 24 h (Figures 2B, 3A). Similar to the 20 nm drug size, the 60 nm drug is distributed in a large volume of tumor tissue during infusion (Figure 2C). The maximum average relative concentration in the tumor tissue is equal to 0.81 at 6 h. Additionally, 6 h after initial injection, the drug is dispersed in the same way as the 20 nm drug but the average relative concentration of drug in the tumor is higher during the whole period (Figure 3A).

### Effect of Tumor Hydraulic Conductivity on Intra-Tumoral Drug Distribution

**FIGURE 4:**
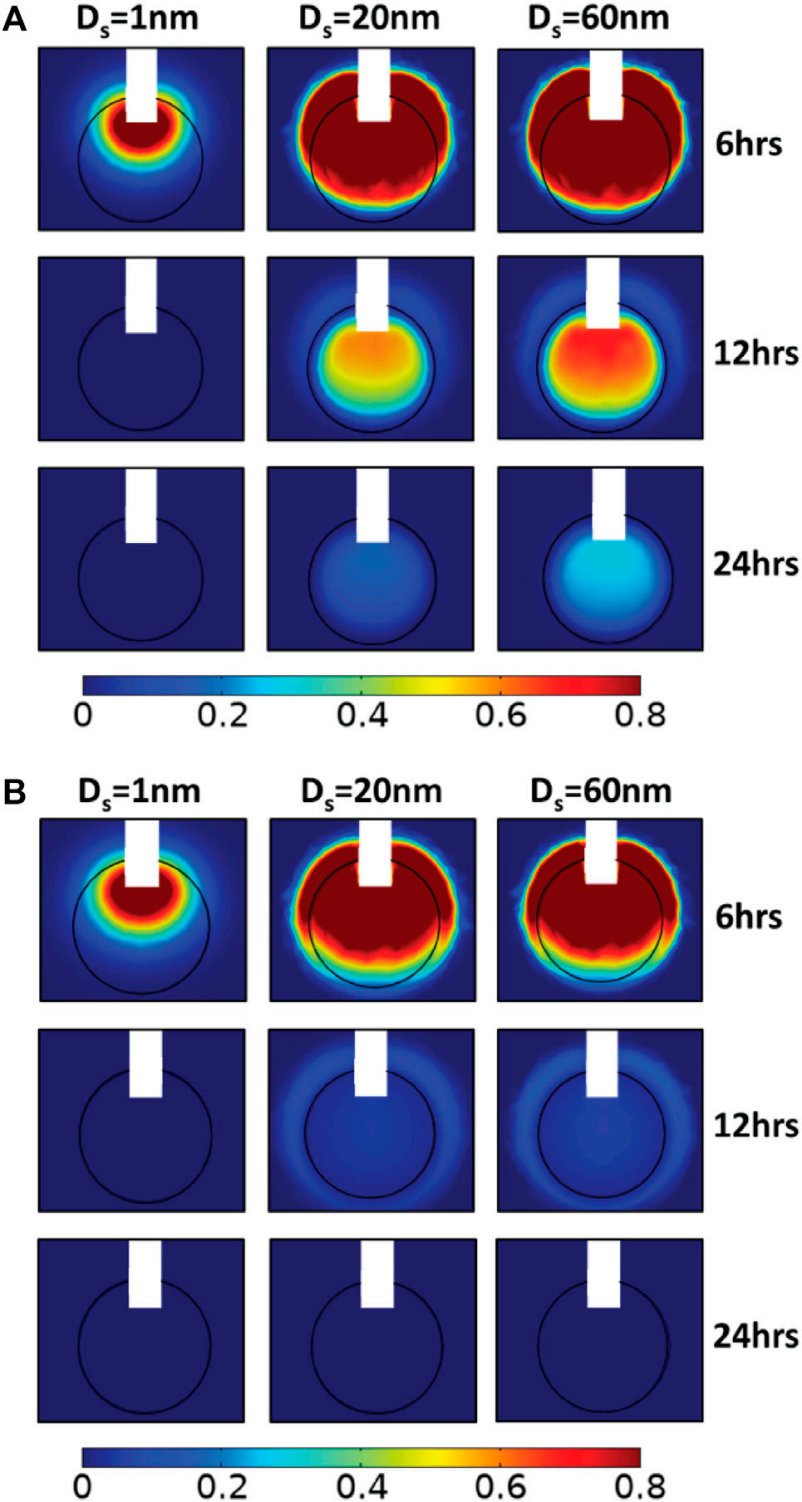
Simulated drug concentration with different hydraulic conductivities of the tumor interstitial space. A sagittal view in the center of tumor tissue showing the spatial distribution of drug concentration for 100 nm pore diameter of vascular walls and for different diameters of the therapeutic agent, D_s_: 1, 20, and 60 nm (columns) in three snapshot: 6, 12, and 24 h (rows), and for different hydraulic conductivities of the tumor interstitial space, *k*: **(A)** 2 × 10^−14^ m^2^/(Pa s) and **(B)** 2 × 10^−12^ m^2^/(Pa s). Drug concentration is normalized by division with the reference value entering the catheter.

The hydraulic conductivity of the tumor describes the resistance to interstitial fluid flow through the pores of the interstitial space of the tissue and thus, it is directly related to IFV and the convective transport of drugs. We repeated simulations to investigate the effect of hydraulic conductivity of the tumor interstitial space on drug concentration and spatial distribution. To achieve this, the hydraulic conductivity was decreased from 2 × 10^−13^ to 2 × 10^−14^ m^2^/(Pa s), whereas the pore diameter of tumor vessel walls was kept to the baseline value of 100 nm. This modification affects insignificantly the spatial distribution of the 1 nm drug (Figures 4A, 3B). In the case of the 20 and 60 nm drugs, higher drug concentrations are observed in the tumor center after infusion (Figure 4A). This can be justified by the IFP and IFV profiles (Supplementary Figure S2). As the hydraulic conductivity of the tumor interstitial space decreases, the velocity within the tumor decreases, while the fluid pressure increases in the tumor center. Therefore, drug transport through convection is reduced in the tumor center, rendering diffusion the dominant transport mechanism, which is inversely proportional to the size of the drug. The convection contribution increases near the tumor boundary, where the drug concentration is low. The maximum drug concentration is located both in the center of the tumor tissue and near the catheter outlet and the drug spreads sufficiently within the tumor tissue. For 20 and 60 nm drug diameter, the average relative concentration values after 12 h are 0.32 and 0.39, respectively, ∼78% greater with respect to the baseline conditions (Figure 3B).

Subsequently, we increased the value of the tumor tissue hydraulic conductivity to 2 × 10^−12^ m^2^/(Pa s), an order of magnitude higher than baseline value. By increasing the hydraulic conductivity of the tumor tissue, the IFP decreases and the IFV rises within the tumor tissue. Thus, convective drug transport in the tumor interstitium is enhanced from the center towards the tumor periphery, allowing the drug to escape easily from tumor tissue. Small molecule drugs with diameters up to 1 nm behave similar to previous simulations as they still diffuse fast, and their transport does not depend on pressure gradients. However, after CED administration, the concentration of all drug sizes is at low levels inside the tumor (Figure 4B). The distributions of nanoparticles with 20 and 60 nm diameters are identical, and the average concentrations decrease sharply as a function of time (Figure 3C). Specifically, after 12 h, the average relative concentration values in the tumor for 20 and 60 nm drugs are 0.048 and 0.056 respectively, ∼74% lower with respect to the baseline conditions.

### Effect of Tumor Vascular Wall Pore Size on Intra-Tumoral Drug Distribution

**FIGURE 5:**
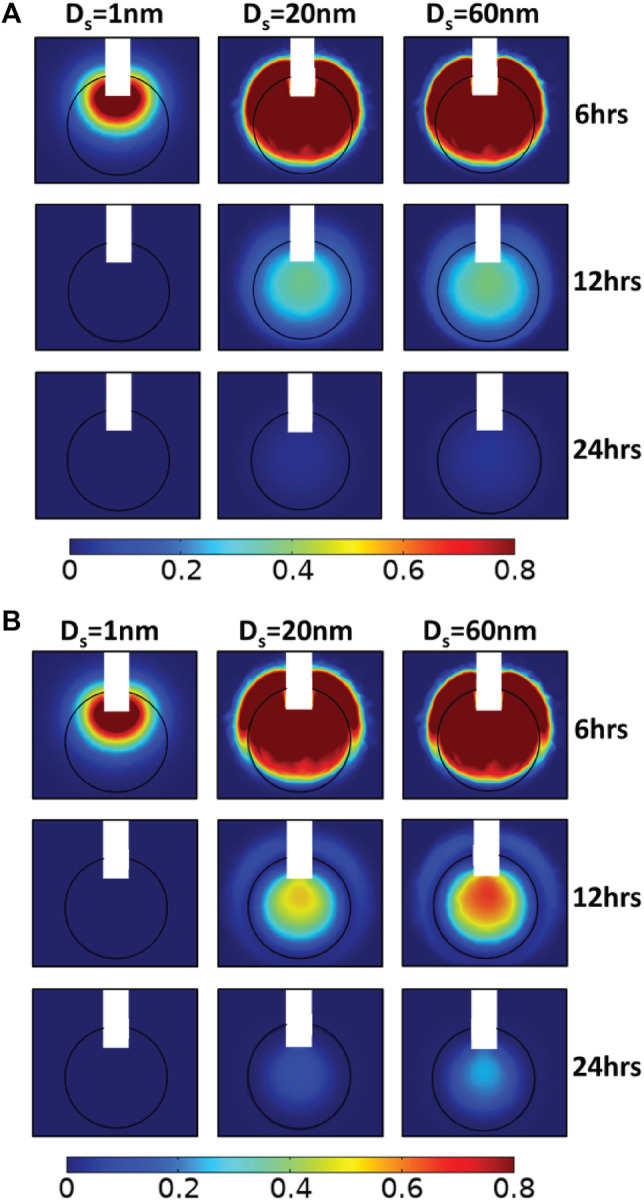
Simulated drug concentration with different vessel wall pore diameters. A sagittal view in the center of tumor tissue showing the spatial distribution of drug concentration for 2 × 10^−13^ m^2^/(Pa s) hydraulic conductivity of the tumor interstitial space, for different diameters of the therapeutic agent, D_s_: 1, 20, and 60 nm in three snapshot: 6, 12, and 24 h, and for different pore diameters of tumor vessel walls, D_o_: **(A)** 50 nm and **(B)** 150 nm. Drug concentration is normalized by division with the reference value entering the catheter.

**FIGURE 6:**
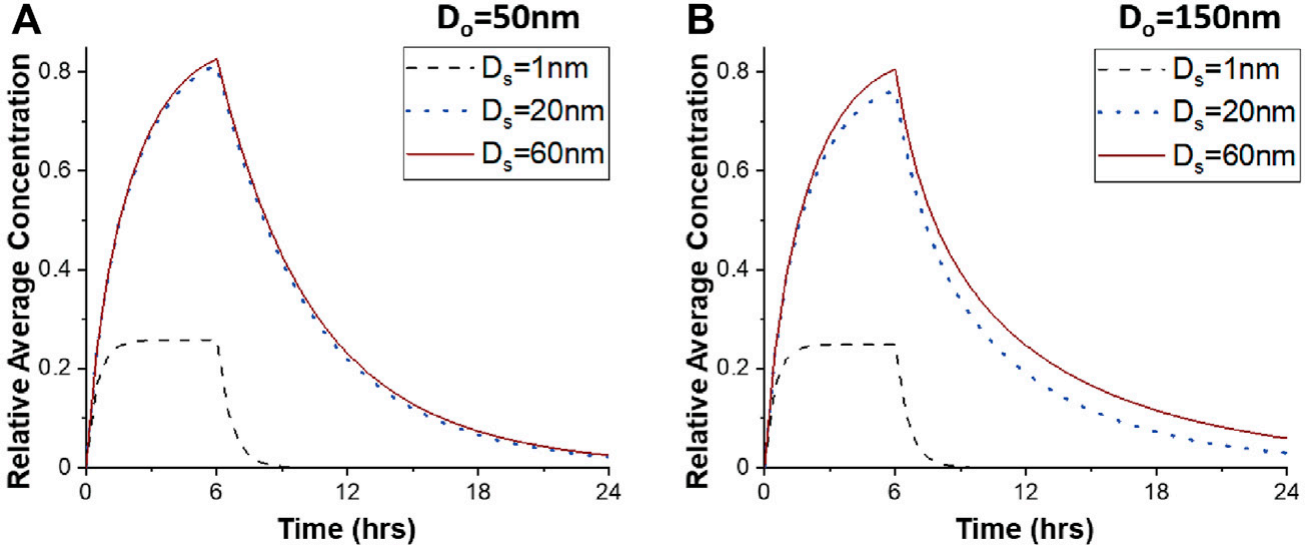
Average drug concentration as a function of the time for different vessel wall pore diameters. Average relative drug concentration in tumor tissue as a function of time for different diameters of therapeutic agents, D_s_, for 2 × 10^−13^ m^2^/(Pa s) hydraulic conductivity of the tumor interstitial space, and for different pore diameters of tumor vascular walls, D_o_: **(A)** 50 nm and **(B)** 150 nm. Drug concentration is normalized by division with the reference value entering the catheter.

Next, we investigated the effect of the pore size of the tumor vessel walls on the distribution of drugs administered through CED. A series of simulations was performed varying the pore diameters of tumor vessel walls and calculated the spatiotemporal distribution of the drugs. The drug diameter range remained the same with the previous simulations and the hydraulic conductivity of tumor tissue was set to 2 × 10^−13^ m^2^/(Pa s). By changing the pore size of vessel walls from 50 to 150 nm, the hydraulic conductivity of the vessels increases considerably, according to Eq. 6. As a result, the fluid pressure increases uniformly inside the tumor and drops steeply at the tumor margin, whereas the velocity magnitude increases at the periphery following the IFP gradients (Supplementary Figure S3), which is a hallmark of tumor patho-physiology (Baxter and Jain, 1989). Taken together, this means that increasing the pore diameter of the vessel walls reduces drug transport through convection. Therefore, the main transport mechanism is through diffusion. Comparing the drug distribution with the baseline tumor microenvironment conditions (Figure 2), the modification of pore dimeters does not influence the spatial distribution of small drugs up to 1 nm diameter (Figure 5). However, the distribution of nanoparticles of 20 and 60 nm diameter differ significantly with respect to the tumor vessel pore size. In the case of 50 nm diameter endothelial wall pores, after infusion, the drug disperses homogeneously within the tumor tissue due to enchanted convection (Figure 5A). Furthermore, it is observed higher local drug concentration for 150 nm pore diameter (Figure 5B). After 12 h, as shown in Figure 6, the average relative concentration for 60 nm drug size is 0.25% ∼14% greater with respect to the baseline conditions.

Supplementary plots for each drug size presenting the average relative drug concentration as a function of time for different values of the hydraulic conductivity of the tissue interstitial space and for different diameters of vessel wall pores are presented in Supplementary Figures S4–S6. These comparative plots show separately the effect of tumor microenvironment properties on the average relative concentration for each drug.

### Heterogeneity of Drug Distribution Within Tumor Site

**FIGURE 7:**
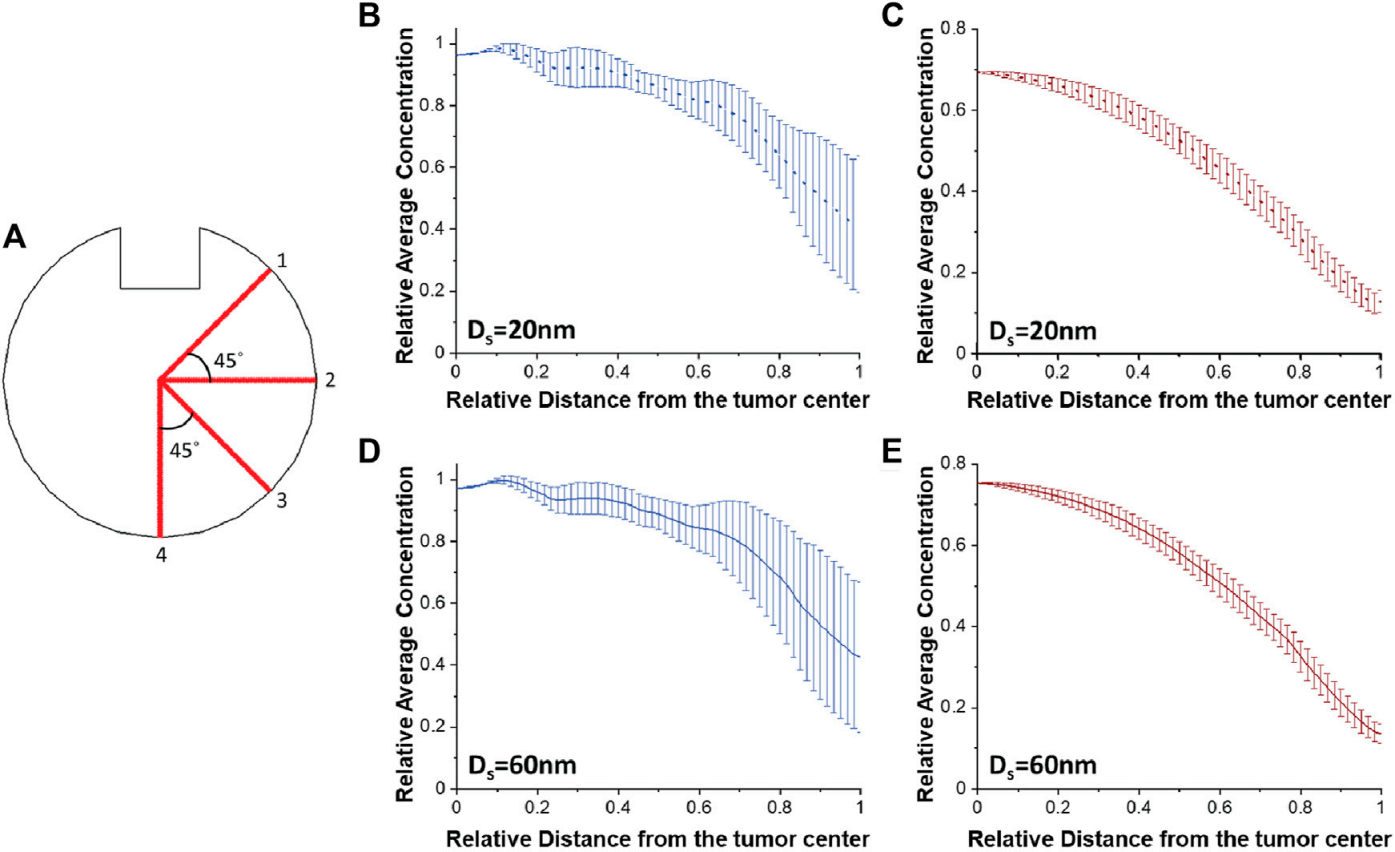
Heterogeneity of drug distribution within tumor tissue. **(A)** Schematic representation of four different directions in the plane for calculating both the average concentration of the drug and the standard error. Plots of the average concentration calculated along the four directions as a function of the distance from the tumor center **(B,D)** after 5 h and **(C,E)** after 9 h, for baseline tumor microenvironment conditions, and for different drug diameters: **(B,C)** 20 nm and **(D,E)** 60 nm. Drug concentration is normalized by division with the reference value entering the catheter. Distance is normalized by division with the tumor radius.

To investigate the heterogeneity of the drug distribution, the drug concentration as a function of distance from the tumor center was calculated for four different directions in the plane (Figure 7A) and the mean value as well as the standard error of the results along these four directions are presented (Figures 7B–E). The standard error bars represent the heterogeneity of the drug distribution, giving a quantification of how close the results are to the average value, i.e., variation of average value. The smaller the error bars, the more homogeneous the distribution of the drug is. The heterogeneity of the drug distribution was assessed during and after CED administration, taking snapshots at 5 and 9 h. The drugs with a diameter of 20 and 60 nm were selected for the heterogeneity measurements because the concentration of the 1 nm drug decreases sharply to zero after infusion. Also, the baseline tumor microenvironment conditions, i.e., 100 nm pore diameter of tumor vessel walls and a 2 × 10^−13^ m^2^/(Pa s) hydraulic conductivity of tumor interstitial space were assumed.

According to the error bars, there is a homogeneous spatial distribution of the drug near the tumor center for both drug diameters during infusion. The heterogeneity exists radially outwards where the magnitude of the error bars increases (Figures 7B,D). This happens because lines 1 and 2 are closer to the infusion site and the drug concentration values are higher along their lines compared to lines 3 and 4 (Figure 7A). After infusion, the average concentrations for both drug sizes gradually decrease as a function of the distance and the spatial distribution of the drug becomes homogeneous (Figures 7C,E), as it is also verified from Figures 2B,C. Furthermore, we repeated simulations varying the tumor hydraulic conductivity (Supplementary Figures S7, S8) with the results following the same patterns as in Figure 7.

### Tumor Drug Accumulation With Catheter Placement Outside of the Tumor

**FIGURE 8:**
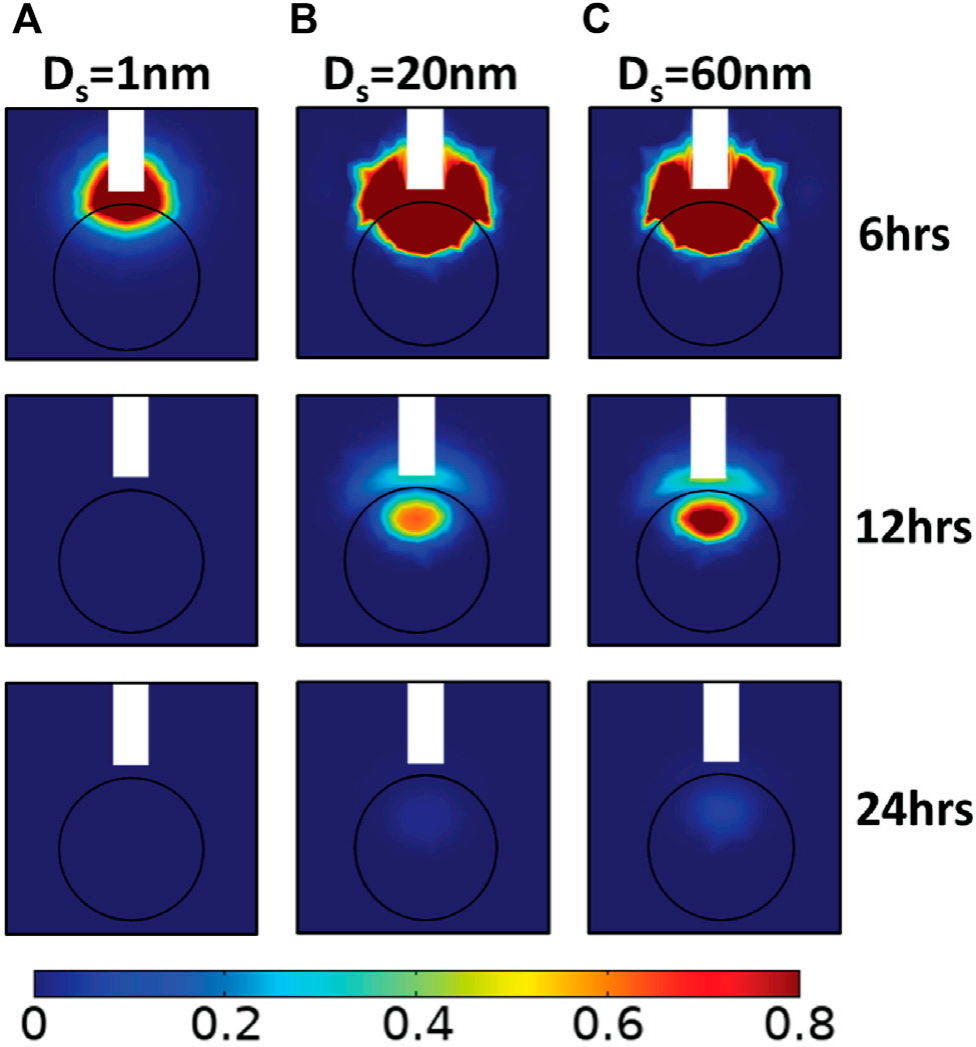
Simulated drug concentration with catheter placement outside the lesion. A sagittal view in the center of tumor showing the spatial distribution of drug concentration for 2 × 10^-13^ m^2^/(Pa s) hydraulic conductivity of the tumor interstitial space, 100 nm diameter of vascular wall pores, and for different therapeutic agents in terms of their hydrodynamic radius, D_s_: **(A)** 1 nm, **(B)** 20 nm, and **(C)** 60 nm, at three snapshots after initial CED injection. Drug concentration is normalized by division with the reference value entering the catheter.

Finally, we investigated the misplacement of the CED catheter. In contrast to the previous simulations where the catheter injects the drug directly into the tumor tissue, the following series of simulations were performed to examine the case that the catheter was taken to be outside of the tumor tissue. To investigate the effect of the catheter placement on drug delivery, the CED catheter was placed 1 mm outside the tumor, as illustrated in Figure 8. During infusion, drug delivery into the tumor is hindered by the pressure gradients at the tumor/heathy tissue interface that drives fluid flow from the tumors towards the healthy tissue. Therefore, high concentrations are found in the healthy tissue. In the case of drug with 1 nm, a tumor volume percentage for relative concentration above 0.2 is approximately 7.5% at the end of the infusion period. For larger drug sizes, after infusion, drug concentration remains high in the tumor tissue. Specifically, after 12 h, the upper part of tumor tissue has 0.8 relative drug concentration and the tumor volume percentage for relative concentration above 0.2 is 12.5% for the 60 nm therapeutic agent.

## Discussion

The present *in silico* work proposed a3D FE model of CED for the treatment of brain cancer. We reported here a parametric *in silico* study of the drug distribution during CED under various drug physicochemical properties and patho-physiological conditions, in terms of vascular wall morphology and tissue properties. To achieve this, we investigated the effect of three crucial parameters on the distribution of the drug, i.e., the therapeutic agent’s size, the hydraulic conductivity of the tumor interstitial space and the pore diameter of the tumor vessel walls. Regarding the drug distribution, the maximum average concentration of the drugs occurs at 6 h at the time that infusion flow stops. The spatial distribution and the average concentration of small drugs of 1 nm diameter are not affected by patho-physiological characteristics of the tumor tissue but they can be significantly affected for nanoparticles larger than 20 nm. When considering high values of the hydraulic conductivity of the tumor interstitial space and for 100 nm pore diameter of vascular walls, the average drug concentration is not sensitive to changes in the size of the drug (effective diameter ranging from 20 to 60 nm; Figure 3). Furthermore, the *in silico* prediction for a drug with 60 nm diameter are promising and remarkable for low values of the hydraulic conductivity of the tumor interstitial space (Figure 4A), and for high pore diameters of vascular wall (Figure 5B). Under these tumor microenvironment properties, the drug is evenly distributed within the tumor interstitial space during and after CED administration. Specifically, the average concentration remains at high levels after 12 h with the maximum drug concentration at the tumor center (Supplementary Figure S6), and the drug spatial distribution is homogeneous after CED (Supplementary Figure S7).

When experimental data is difficult to obtain, *in silico* models can provide useful information. Indeed, the *in silico* predictions, provided they are sufficiently validated, can provide further insights into the spatial distribution and the average drug concentration in the tumor. In this contribution, tumor IFP and IFV profiles after CED can be confirmed to relevant literature findings (Baxter and Jain, 1989; Jain et al., 2007). Additionally, the relation between the tumor interstitial fluid pressure and the tumor microenvironment properties is confirmed from previous *in silico* studies (Jain et al., 2007). The range of the tumor interstitial fluid velocity values agrees with the experimental data, which show that the magnitude of fluid velocity varies from 10^−7^ to 10^−6^ m/s in the brain tissue (Fung et al., 1996; Fleming and Saltzman, 2002; Kimelberg, 2004). Nevertheless, *in vivo* measurements of drug distribution in the human brain during CED is an exceptional challenge, thus, rendering *in silico* drug delivery validation difficult. Consequently, verification of the IFP and IFV profiles is essential to confirm the accuracy of the model predictions. Additionally, recent studies performed *in vivo* experiments to investigate the crucial parameters of CED administration (Singleton et al., 2018; Tosi et al., 2019; Brady et al., 2020). It was observed that the capillary loss rates are elevated for small molecules, thus their tumor coverage is much lower (Brady et al., 2020). Our model predictions are qualitatively consistent with these *in vivo* observations, in such that the most promising nanoparticle size could be greater than 10 nm for better CED administration. Moreover, the calculated tumor microenvironment properties, i.e., the vascular permeability of the drug and the hydraulic conductivity of the vascular wall, as well as the applicability of the theory for hindered transport of rigid solutes have been verified in previous research efforts (Hobbs et al., 1998; Dreher et al., 2006; Scallan and Huxley, 2010; Chauhan et al., 2012; Mpekris et al., 2015).

It is important, however, to acknowledge the simplifications and limitations of the present model. For the sake of simplicity, the drug was considered a spherical particle and the vessel wall openings were modeled as perfect cylindrical pores. Therefore, the theory for hindered transport of rigid solutes through liquid filled pores could be applied to describe drug transport across the tumor vessel walls. Additionally, to simplify the modelling procedure, the flow physics of the catheter domain were disregarded. This modelling approach was substantially less challenging, as the complexity (in terms of the extra differential equations and the additional model parameters and boundary conditions) and the considerable computational burden (solving the Navier-Stokes equations and the coupling of these with the biphasic FE formulation) were minimized. The catheter jet flow was modeled by taking appropriate boundary conditions at the interface between the catheter and the (tumor or host) tissue in order to simulate the drug administration during CED. After CED administration, a zero-flux boundary condition was applied on the outlet surface of the catheter, neglecting any drug amount that can be diffused from the catheter to the tumor tissue. Regarding the model limitations, it incorporates only biomechanical properties of the grey and white matter, while it does not account for other components of the brain, such as the thalamus, the internal capsule, the corpus callosum, the putamen, ventricles, and cavities. How the predictive results would be affected by the incorporation of the other brain components is not intuitive and, thus, detailed simulations would have to be performed. Also, according to the literature (Linninger et al., 2008), the drug transport efficiency varies greatly in different regions of the brain, since the effective diffusivity in the gray matter is isotropic, whereas white matter diffusion is anisotropic (Shimony et al., 1999; Cao et al., 2003; Pecheva et al., 2018). Hence, diffusion tensor imaging (DTI) data could be considered to provide estimates of the tissue anisotropy within the entire brain and, therefore, reduce these uncertainties.

To conclude, modifying the tumor microenvironment properties (e.g., by pharmaceutical interventions) prior to the drug administration through CED, may be suitable for effective drug delivery within the tumor, while simultaneously minimizing drug toxicity to the healthy brain tissue. Our *in silico* predictions provide further and useful insights of the spatial distribution and the drug concentration in the tumor towards improving brain cancer therapy. Based on our results, it is predicted that the chemotherapeutics (drug size: 1 nm) are diffused rapidly away from the tumor either through the blood vessels or from the tumor periphery, and thus, their average concentration in the tumor tissue is significantly lower compared to liposomes or other nanoparticles (drug size: >10 nm).

## Data Availability

The raw data supporting the conclusion of this article will be made available by the authors, without undue reservation.
