# Supplementary material for: Convection-Enhanced Delivery In Silico Study for Brain Cancer Treatment
Source: Front Bioeng Biotechnol. 2022 May 25;10:867552. doi: 10.3389/fbioe.2022.867552 (PMC9177080; doi:10.3389/fbioe.2022.867552)
Supplement: Supplementary file 1 [file DataSheet1.pdf]

Supplementary Material

Supplementary Table 1: Comparison summary of previous relevant studies with respect to the CED modelling approach of this work.

	**(Støverud et al., 2012)**	**(Zhan and Wang, 2018)**	**(Brady et al., 2020)**	**Present Study**
Brain tissue model: deformation during CED infusion	poroelastic model<br>• isotropic linear elasticity<br>• small deformations	not included (rigid model)	poroelastic model<br>• isotropic linear elasticity<br>• incompressibility	not included (rigid model)
drug trans-vascular transport through vasculature (tumor/ healthy)	simulated using constant source/sink term for the therapeutic agent (see Eq. (12) therein); adsorption of infused therapeutic agent is neglected.	simulated using constant drainage rate of the free drug from blood vessels (vascular permeability to liposomes/doxorubi cin, microvascular density; see Eq. (6) therein).	simulated using constant rate of the free drug (sum of rates due to capillary loss and to irreversible degradation; see Table 1 therein).	theory for hindered transport of rigid solutes through liquid filled porous; modeled explicitly effect of drug size and vessel walls' pore size (vascular morphology)
CED timescale simulated	during CED infusion (infusion duration: 2 hrs, 12 hrs)	during CED infusion (infusion duration: 24 hrs)	during CED infusion (infusion duration: 24 hrs – 72 hrs)	during and after CED infusion (infusion duration: 6 hrs)
model parameters interrogated in computer simulations	• Tissue permeability<br>• Rigid model *vs* Elastic model<br>• Elastic Properties (Young's modulus, Poisson's ratio)	• Microvascular density<br>• Drug diffusivity, and vascular permeability<br>• Drug release rate<br>• Infusion rate<br>• Liposome solution concentration	• Drug (therapeutic agent or surrogate) size<br>• Infusion protocol *wrt* use of multiple catheters placement<br>• Infusion protocol *wrt* total number of infusions<br>• Infusion rate	• Tissue hydraulic conductivity<br>• Pore size of tumor vessels<br>• Drug (hydrodynamic) size:<br>Drug diffusivity, and vascular permeability
In vivo investigation	included (DTI data from 4 patients to calibrate tissue permeability)	not included	included (CED protocols based on human clinical trials: MR1-1 and D2C7)	not included

Supplementary Table 2: Reflection coefficient and hydrodynamic coefficient definitions.

The reflection coefficient is defined by the expression: $\sigma_f = 1 - W$, and the vascular permeability of the drug, P, is defined by equation (7), where W and H correspond to the hydrodynamic coefficients for neutral spheres in cylindrical pores: $H = 6\,\pi\,F\,{K_t}^{-1}$, and $W = (2 - F)\,F\,K_s\,{K_t}^{-1}/2$, where F is the partition coefficient: $F = (1-\lambda)^2$, λ is the ratio of the drug size to the vessel wall pore size. Coefficients K_t and K_s are calculated through:

$$\begin{Bmatrix} K_t \\ K_s \end{Bmatrix} = \frac{9\,\pi^2}{2\sqrt{2}}(1-\lambda)^{-2.5} + \left[1 + \sum_{n=1}^{2} \begin{Bmatrix} a_n \\ b_n \end{Bmatrix} (1-\lambda)^n\right] + \sum_{n=0}^{4} \begin{Bmatrix} a_{n+3} \\ \mathrm{b}_{n+3} \end{Bmatrix} \lambda^n,$$

where a and b are constant coefficients (Deen, 1987).

**Coefficient a_n**	**Value**	**Coefficient b_n**	**Value**
a_1	−1.217	b_1	0.117
a_2	1.534	b_2	−0.044
a_3	−22.508	b_3	4.018
a_4	−5.617	b_4	−3.979
a_5	−0.336	b_5	−1.922
a_6	−1.216	b_6	4.392
a_7	1.647	b_7	5.006

Supplementary Table 3: Values of all parameters used in the CED brain model.

** Normal tissue includes grey and white matter of the brain*

Parameter	Description	Domain	Value	Reference
r_s	Drug radius	-	0.5, 10, 30 nm	(Koo et al., 2006, Allard et al., 2009)
r_o	Vascular wall pore radius	Normal tissue	3.5 nm	(Hobbs et al., 1998, Sarin et al., 2009, Wang et al., 2011)
		Tumor tissue	25, 50, 75 nm	
D_i	Drug diffusion coefficient	All tissues	5.82×10^{-10} m²/s for 0.5nm drug radius, 9.28×10^{-12} m²/s for 10nm drug radius, 3.28×10^{-12} m²/s for 30nm drug radius	(Pluen et al., 2001)
k	Hydraulic conductivity of tissue interstitial space	Grey Matter	2×10^{-14} m²/(Pa·s)	(Netti et al., 2000, Angeli and Stylianopoulos, 2016, Smith and Humphrey, 2007)
		White Matter	2×10^{-13} m²/(Pa·s)	
		Tumor tissue	2×10^{-14} m²/(Pa·s)<br>2×10^{-13} m²/(Pa·s)<br>2×10^{-13} m²/(Pa·s)	
S_v	Vascular density of blood vessels	Normal tissue	70 (cm)$^{-1}$	(Mpekris et al., 2017, Jain et al., 2007)
		Tumor tissue	50 (cm)$^{-1}$	
$L_{pl}\, S_{vl}$	Permeability of Lymphatics	Normal tissue	2.24×10^{-11} m²·s/kg	(Scallan and Huxley, 2010, Stylianopoulos et al., 2018)
		Tumor tissue	0	
γ	Fraction of vessel wall surface area occupied by pores	All tissues	1×10^{-3}	(Mpekris et al., 2017)
C_v	Vascular drug concentration	All tissues	0	-
p_v	Vascular pressure of blood vessels	All tissues	2.0 kPa	(Stylianopoulos et al., 2018)
p_l	Vascular pressure of lymphatic vessels	All tissues	0.0 kPa	(Voutouri and Stylianopoulos, 2014)
L_{vw}	Vessel wall thickness	All tissues	5×10^{-6} m	(Stylianopoulos et al., 2013)
T	Temperature	All tissues	310 K	(Mpekris et al., 2017)
μ_i	Interstitial fluid viscosity	All tissues	7.8×10^{-4} Pa·s	(Zhan and Wang, 2018,

μ	Plasma viscosity	-	1.30×10^{-3} Pa·s	Kesmarky et al., 2008)
ρ	Interstitial fluid density	All tissues	1000 kg/m³	(Zhan and Wang, 2018)
ε_p	Tissue porosity	All tissues	0.3	(Linninger et al., 2008)
k_d	Drug degradation constant	All tissues	2×10^{-5} s^{-1}	(Linninger et al., 2008)
k_l	Lymphatic drainage constant	Normal tissue	1×10^{-4} s^{-1}	(Zhan et al., 2018)

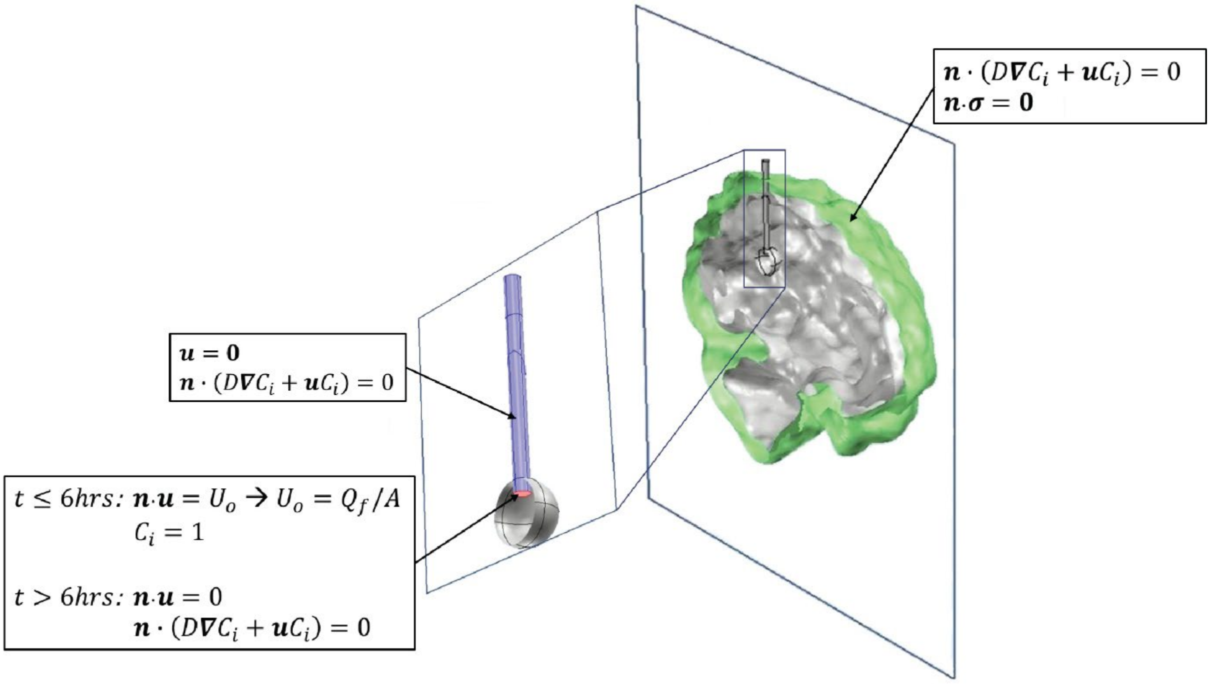

Supplementary Figure 1. Boundary conditions of the 3D model. At the interface between the catheter and tumor the normal inlet velocity (*i.e.,* $U_o=Q_f/A$, A cross section of the catheter) was taken equal to 1.99×10^{-5}m/s and infusion lasted for 6 hours. Also, at the interface of the catheter and tumor tissue, the relative drug concertation was set to unity for the period of the infusion and after completion of infusion, a zero-flux boundary condition was applied (*i.e.*, $\boldsymbol{n}\cdot(D\nabla C_i + \boldsymbol{u}\, C_i)=0$, where $\boldsymbol{n}$ corresponds to the outward unit normal vector).The normal stresses on the outer brain surfaces were equal to zero (*i.e.*, $\boldsymbol{n}\cdot\boldsymbol{\sigma} = \boldsymbol{0}$). At the catheter surfaces, a no-slip boundary condition was applied for fluid velocity (*i.e.*, $\boldsymbol{u}=\boldsymbol{0}$) and additionally, a zero-flux boundary condition was set for the transport of the drugs. The latter boundary condition was also set at the outer brain surfaces.

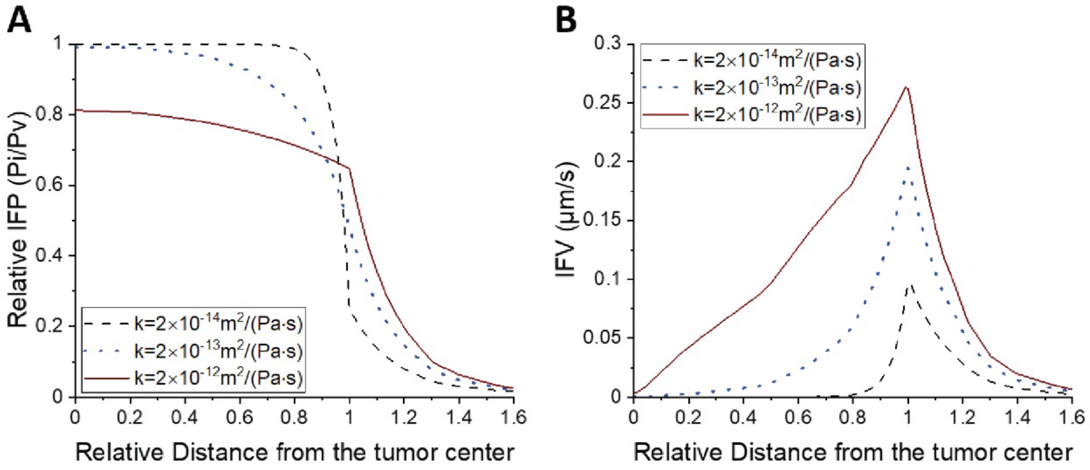

Supplementary Figure 2. IFP and IFV as a function of the distance from the tumor center for baseline value of the vessel wall pore diameter. IFP and IFV as a function of the distance from the tumor center for different hydraulic conductivities of the tumor interstitial space and 100 nm diameter of vascular wall pores. *In silico* predicted **(A)** relative fluid pressure (p_i/p_v) and **(B)** velocity magnitude of the tumor interstitial space before CED administration. IFP is normalized by division with the vascular pressure of blood vessels, p_v. Distance is normalized by division with the tumor radius.

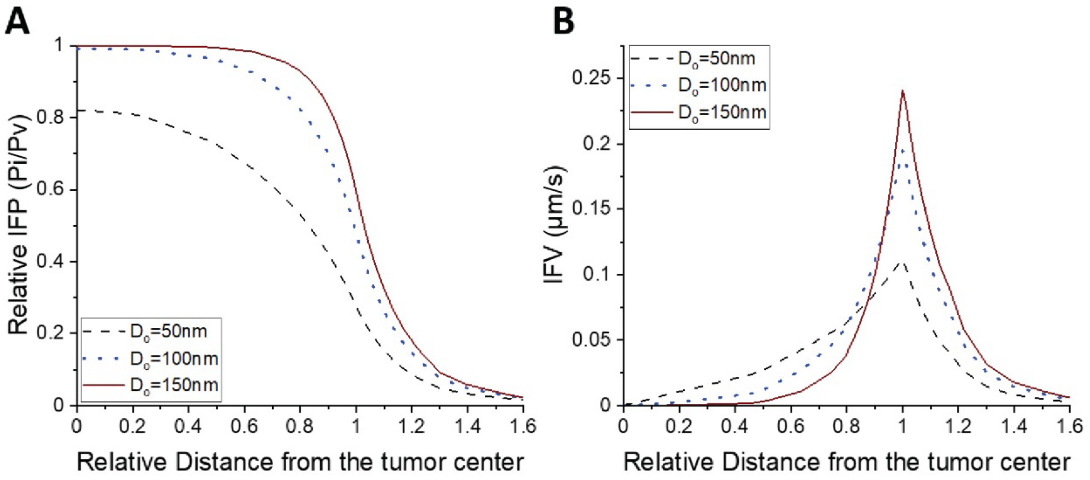

Supplementary Figure 3. IFP and IFV as a function of the distance from the tumor center for baseline value of hydraulic conductivity of the tumor interstitial space. IFP and IFV as a function of the distance from the tumor center for different diameters of vascular wall pores and 2×10^{-13} $m^2/(Pa\cdot s)$ hydraulic conductivity of the tumor interstitial space. *In silico* predicted **(A)** relative fluid pressure (p_i/p_v) and **(B)** velocity magnitude of the tumor interstitial space before CED administration. IFP is normalized by division with the vascular pressure of blood vessels, p_v. Distance is normalized by division with the tumor radius.

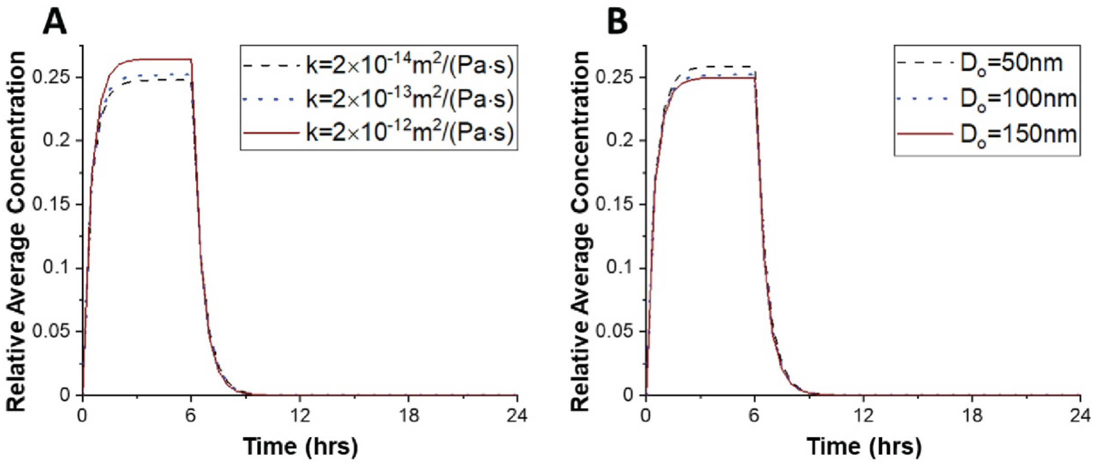

Supplementary Figure 4. Average concentration in tumor tissue as a function of time for 1 nm diameter of the therapeutic agent. *In silico* predicted average relative concentration: **(A)** different hydraulic conductivities of the tumor interstitial space and 100 nm diameter of vascular wall pores and **(B)** different diameters of vascular wall pores and 2×10^{-13} $m^2/(Pa{\cdot}s)$ hydraulic conductivity of the tumor interstitial space. Drug concentration is normalized by division with the reference value entering the catheter.

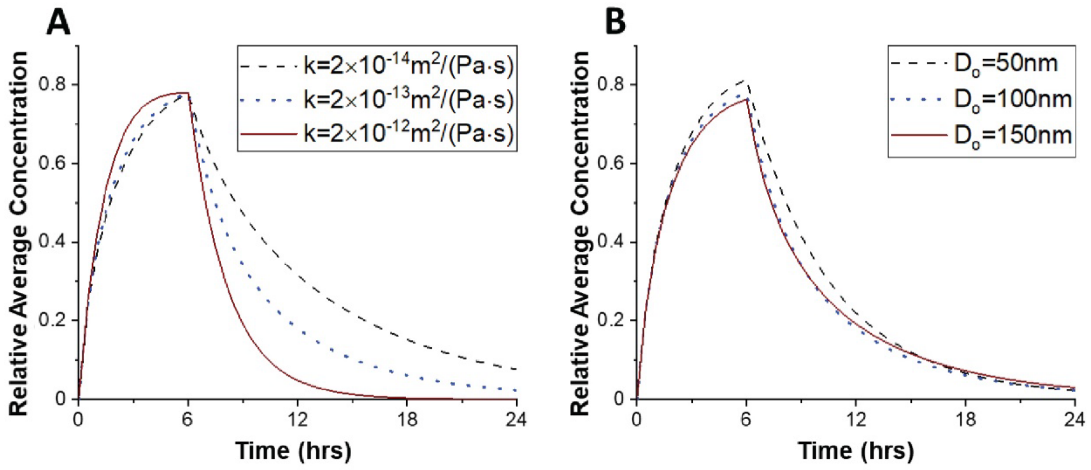

Supplementary Figure 5. Average concentration in tumor tissue as a function of the time for 20 nm diameter of the therapeutic agent. *In silico* predicted average relative concentration: **(A)** different hydraulic conductivities of the tumor interstitial space and 100 nm diameter of vascular wall pores and **(B)** different diameters of vascular wall pores and $2\times10^{-13} m^2/(Pa{\cdot}s)$ hydraulic conductivity of the tumor interstitial space. Drug concentration is normalized by division with the reference value entering the catheter.

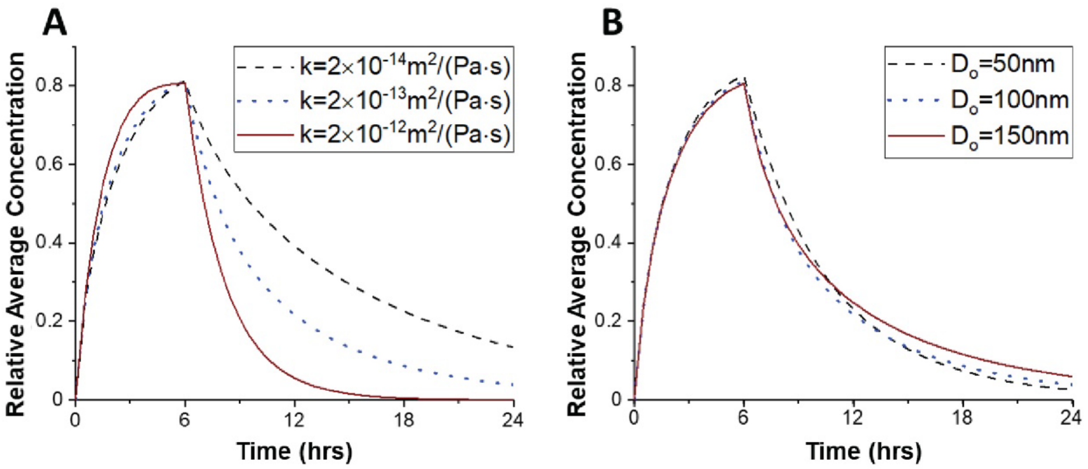

Supplementary Figure 6. Average concentration in tumor tissue as a function of the time for 60 nm diameter of the therapeutic agent. *In silico* predicted average relative concentration: **(A)** different hydraulic conductivities of the tumor interstitial space and 100 nm diameter of vascular wall pores and **(B)** different diameters of vascular wall pores and 2×10^{-13} $m^2/(Pa{\cdot}s)$ hydraulic conductivity of the tumor interstitial space. Drug concentration is normalized by division with the reference value entering the catheter.

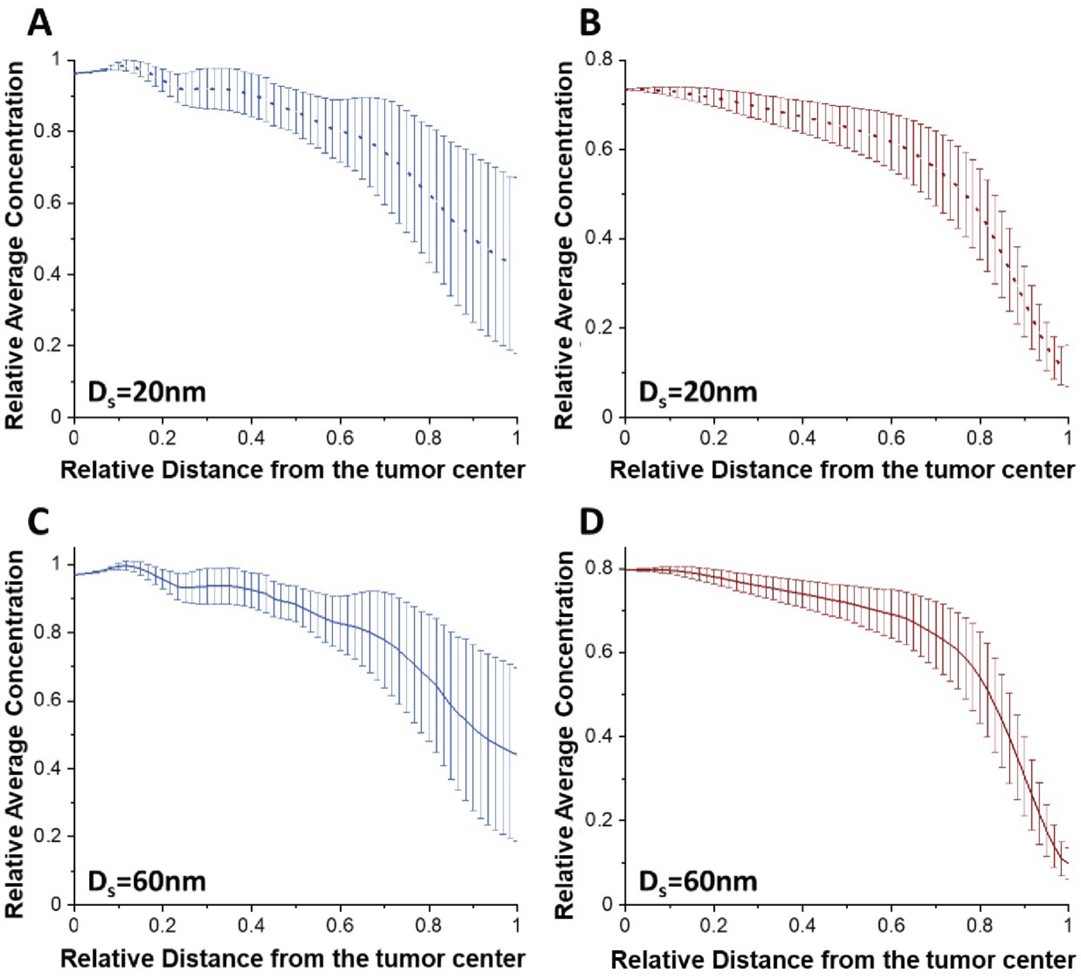

Supplementary Figure 7. Average drug concentration as a function of the distance from the tumor center plots for the lowest hydraulic conductivity of the tumor interstitial space. Average relative concentration calculated along the four directions as a function of the relative distance from the tumor center **(A, C)** after 5 hrs and **(B, D)** after 9 hrs, for 2×10^{-14} $m^2/(Pa{\cdot}s)$ hydraulic conductivity of the tumor interstitial space, 100 nm different pore diameter of tumor vascular walls, and for different drug diameters: **(A, B)** 20 nm and **(C, D)** 60 nm. Drug concentration is normalized by division with the reference value entering the catheter. Distance is normalized by division with the tumor radius.

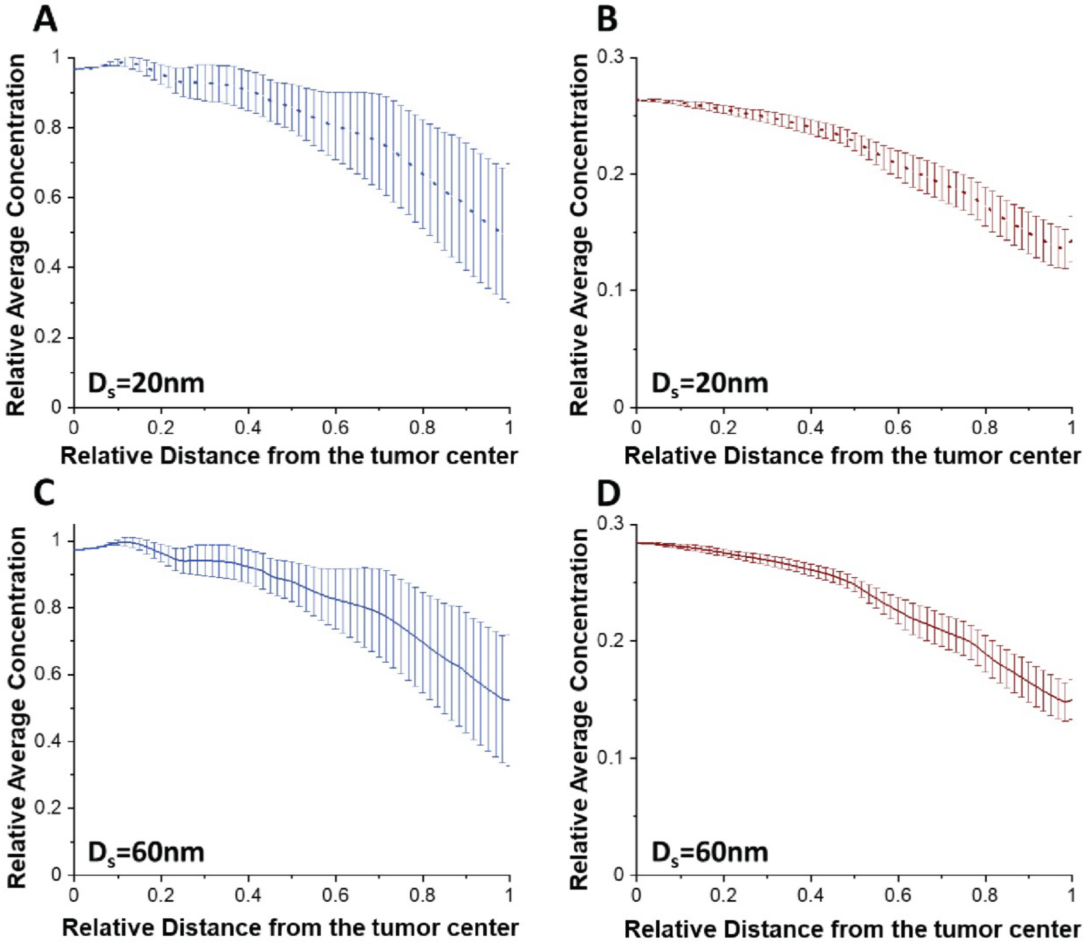

Supplementary Figure 8. Average drug concentration as a function of the distance from the tumor center plots for the highest hydraulic conductivity of the tumor interstitial space. Average relative concentration calculated along the four directions as a function of the relative distance from the tumor center **(A, C)** after 5 hrs and **(B, D)** after 9 hrs, for 2×10^{-12} $m^2/(Pa{\cdot}s)$ hydraulic conductivity of the tumor interstitial space, 100 nm different pore diameter of tumor vascular walls, and for different drug diameters: **(A, B)** 20 nm and **(C, D)** 60 nm. Drug concentration is normalized by division with the reference value entering the catheter. Distance is normalized by division with the tumor radius.

References

ALLARD, E., PASSIRANI, C. & BENOIT, J. P. 2009. Convection-enhanced delivery of nanocarriers for the treatment of brain tumors. *Biomaterials,* 30, 2302-18.

ANGELI, S. & STYLIANOPOULOS, T. 2016. Biphasic modeling of brain tumor biomechanics and response to radiation treatment. *J Biomech*.

BRADY, M., RAGHAVAN, R. & SAMPSON, J. 2020. Determinants of Intraparenchymal Infusion Distributions: Modeling and Analyses of Human Glioblastoma Trials. *Pharmaceutics,* 12.

DEEN, W. M. 1987. Hindered Transport of Large molecules in Liquid-Filled Pores. *AIChE J,* 33, 1409-1425.

HOBBS, S. K., MONSKY, W. L., YUAN, F., ROBERTS, W. G., GRIFFITH, L., TORCHILIN, V. P. & JAIN, R. K. 1998. Regulation of transport pathways in tumor vessels: role of tumor type and microenvironment. *Proc Natl Acad Sci U S A,* 95, 4607-12.

JAIN, R. K., TONG, R. T. & MUNN, L. L. 2007. Effect of vascular normalization by antiangiogenic therapy on interstitial hypertension, peritumor edema, and lymphatic metastasis: insights from a mathematical model. *Cancer Res,* 67, 2729-35.

KESMARKY, G., KENYERES, P., RABAI, M. & TOTH, K. 2008. Plasma viscosity: a forgotten variable. *Clin Hemorheol Microcirc,* 39, 243-6.

KOO, Y.-E. L., REDDY, G. R., BHOJANI, M., SCHNEIDER, R., PHILBERT, M. A., REHEMTULLA, A., ROSS, B. D. & KOPELMAN, R. 2006. Brain cancer diagnosis and therapy with nanoplatforms. *Advanced drug delivery reviews,* 58, 1556-1577.

LINNINGER, A. A., SOMAYAJI, M. R., MEKARSKI, M. & ZHANG, L. 2008. Prediction of convection-enhanced drug delivery to the human brain. *J Theor Biol,* 250, 125-38.

MPEKRIS, F., BAISH, J. W., STYLIANOPOULOS, T. & JAIN, R. K. 2017. Role of vascular normalization in benefit from metronomic chemotherapy. *Proc Natl Acad Sci U S A,* 114, 1994-1999.

NETTI, P. A., BERK, D. A., SWARTZ, M. A., GRODZINSKY, A. J. & JAIN, R. K. 2000. Role of extracellular matrix assembly in interstitial transport in solid tumors. *Cancer Res,* 60, 2497-503.

PLUEN, A., BOUCHER, Y., RAMANUJAN, S., MCKEE, T. D., GOHONGI, T., DI TOMASO, E., BROWN, E. B., IZUMI, Y., CAMPBELL, R. B., BERK, D. A. & JAIN, R. K. 2001. Role of tumor-host interactions in interstitial diffusion of macromolecules: cranial vs. subcutaneous tumors. *Proc Natl Acad Sci U S A,* 98, 4628-33.

SARIN, H., KANEVSKY, A. S., WU, H., SOUSA, A. A., WILSON, C. M., ARONOVA, M. A., GRIFFITHS, G. L., LEAPMAN, R. D. & VO, H. Q. 2009. Physiologic upper limit of pore size in the blood-tumor barrier of malignant solid tumors. *J Transl Med,* 7, 51.

SCALLAN, J. P. & HUXLEY, V. H. 2010. In vivo determination of collecting lymphatic vessel permeability to albumin: a role for lymphatics in exchange. *J Physiol,* 588, 243-54.

SMITH, J. H. & HUMPHREY, J. A. 2007. Interstitial transport and transvascular fluid exchange during infusion into brain and tumor tissue. *Microvasc Res,* 73, 58-73.

STØVERUD, K. H., DARCIS, M., HELMIG, R. & HASSANIZADEH, S. M. 2012. Modeling concentration distribution and deformation during convection-enhanced drug delivery into brain tissue. *Transport in porous media,* 92, 119-143.

STYLIANOPOULOS, T., MARTIN, J. D., SNUDERL, M., MPEKRIS, F., JAIN, S. R. & JAIN, R. K. 2013. Coevolution of solid stress and interstitial fluid pressure in tumors during progression: Implications for vascular collapse. *Cancer research,* 73, 3833-3841.

STYLIANOPOULOS, T., MUNN, L. L. & JAIN, R. K. 2018. Reengineering the Physical Microenvironment of Tumors to Improve Drug Delivery and Efficacy: From Mathematical Modeling to Bench to Bedside. *Trends Cancer,* 4, 292-319.

VOUTOURI, C. & STYLIANOPOULOS, T. 2014. Evolution of osmotic pressure in solid tumors. *J Biomech,* 47, 3441-7.

WANG, J., LU, Z., GAO, Y., WIENTJES, M. G. & AU, J. L. 2011. Improving delivery and efficacy of nanomedicines in solid tumors: role of tumor priming. *Nanomedicine (Lond),* 6, 1605-20.

ZHAN, W., ALAMER, M. & XU, X. Y. 2018. Computational modelling of drug delivery to solid tumour: Understanding the interplay between chemotherapeutics and biological system for optimised delivery systems. *Adv Drug Deliv Rev,* 132**,** 81-103.

ZHAN, W. & WANG, C. H. 2018. Convection enhanced delivery of liposome encapsulated doxorubicin for brain tumour therapy. *J Control Release,* 285**,** 212-229.
